# Can you hear me now? Range‐testing a submerged passive acoustic receiver array in a Caribbean coral reef habitat

**DOI:** 10.1002/ece3.2228

**Published:** 2016-06-17

**Authors:** Thomas H. Selby, Kristen M. Hart, Ikuko Fujisaki, Brian J. Smith, Clayton J. Pollock, Zandy Hillis‐Starr, Ian Lundgren, Madan K. Oli

**Affiliations:** ^1^ Fort Lauderdale Research and Education Center University of Florida 3205 College Avenue Davie Florida 33314; ^2^ Wetland and Aquatic Research Center United States Geological Survey 3321 College Avenue Davie Florida 33314; ^3^ National Park Service Buck Island Reef National Monument Christiansted St. Croix 008020‐4611; ^4^ Naval Facilities Engineering Command Pacific, 258 Makalapa Dr, Suite 100 Honolulu Hawaii 96860‐3139; ^5^ Department of Wildlife Ecology and Conservation University of Florida Newins‐Ziegler Hall Gainesville Florida 32611‐0430

**Keywords:** Caribbean reef, passive acoustic telemetry, range‐testing, VR2W

## Abstract

Submerged passive acoustic technology allows researchers to investigate spatial and temporal movement patterns of many marine and freshwater species. The technology uses receivers to detect and record acoustic transmissions emitted from tags attached to an individual. Acoustic signal strength naturally attenuates over distance, but numerous environmental variables also affect the probability a tag is detected. Knowledge of receiver range is crucial for designing acoustic arrays and analyzing telemetry data. Here, we present a method for testing a relatively large‐scale receiver array in a dynamic Caribbean coastal environment intended for long‐term monitoring of multiple species. The U.S. Geological Survey and several academic institutions in collaboration with resource management at Buck Island Reef National Monument (BIRNM), off the coast of St. Croix, recently deployed a 52 passive acoustic receiver array. We targeted 19 array‐representative receivers for range‐testing by submersing fixed delay interval range‐testing tags at various distance intervals in each cardinal direction from a receiver for a minimum of an hour. Using a generalized linear mixed model (GLMM), we estimated the probability of detection across the array and assessed the effect of water depth, habitat, wind, temperature, and time of day on the probability of detection. The predicted probability of detection across the entire array at 100 m distance from a receiver was 58.2% (95% CI: 44.0–73.0%) and dropped to 26.0% (95% CI: 11.4–39.3%) 200 m from a receiver indicating a somewhat constrained effective detection range. Detection probability varied across habitat classes with the greatest effective detection range occurring in homogenous sand substrate and the smallest in high rugosity reef. Predicted probability of detection across BIRNM highlights potential gaps in coverage using the current array as well as limitations of passive acoustic technology within a complex coral reef environment.

## Introduction

Researching the movement patterns and behavior of highly mobile marine vertebrates is challenging (Hyrenbach et al. 2000; Sale et al. 2005). Technological advances with GPS, satellite, and other bio‐logging devices have provided data on large‐scale movement patterns for numerous species (Ropert‐Coudert and Wilson 2005; Hooker et al. 2007; Hart and Hyrenbach 2009). However, these tools are often expensive, sometimes require manual retrieval, or offer only coarse‐resolution data (Hazel et al. 2013). Submerged passive acoustic telemetry is an increasingly popular technology being used to investigate fine‐scale movement patterns of aquatic species (Heupel et al. 2006; Kessel et al. 2014b). The use of passive acoustic technology in ecological studies is expected to increase as technological advances reduce costs for existing equipment, making larger, more complex arrays financially feasible (Heupel et al. 2006; Hussey et al. 2015).

Passive acoustic tracking uses a submersible receiver paired with an omnidirectional hydrophone in a single unit referred to simply as a receiver, to “listen” continually for acoustic pulses emitted at a certain frequency from tags implanted or attached to an organism of interest. Acoustic signals within detection range of the receiver are decoded and the corresponding transmitter identity (ID) recorded, along with the date and time the detection occurred. Several companies manufacture passive acoustic equipment; for this study, we used Vemco VR2W receivers and V16 and V13 range‐testing tags (Vemco 2014).

VR2Ws or similar acoustic receivers can facilitate long‐term monitoring of highly mobile marine species as they allow for continuous tracking of multiple species over extended periods of time. The configuration and size of acoustic receiver arrays depend on the research question and habitat features where the study is being conducted. Early research utilizing passive acoustic technology was limited to a few isolated receivers to investigate the presence or absence of tagged individuals (*e.g*., Klimley & Nelson, 1984). With decreasing costs and technological advances (*e.g*., battery miniaturization), receivers and transmitters are now being deployed in a variety of different contexts (Kessel et al. 2014b). Three or more receivers with overlapping detection ranges can allow for fine‐scale calculation of tag locations to within <5 m (Heupel et al. 2006). Overlapping detection ranges can also allow for gates or curtains typically in physically constrained environments, such as rivers or estuaries, to help elucidate movement to and from an area (Stark et al. 2005). Numerous isolated receivers can be deployed in a grid or irregular array formation to cover relatively large open geographic areas such as marine protected areas (Chapman et al. 2005; Kerwath et al. 2009; O'Toole et al. 2011; Garcia et al. 2014). Collaborations among researchers using acoustic receivers, such as the Florida Atlantic Coast Telemetry (FACT) group, make region‐wide studies of highly mobile species feasible (Kessel et al. 2014a; Pittman et al. 2014; Reyier et al. 2014; Young et al. 2014).

In order to effectively employ passive acoustic telemetry for ecological research, researchers need to understand the relationship between the probability of detecting a tag at different distances from the receiver and the factors potentially influencing that relationship (hereafter, “range‐testing”). Environmental variables at receiver locations can cause heterogeneity in effective receiver detection range (*i.e*., the maximum distance at which ≥50.0% of detections are successfully recorded) even within a relatively small array (Payne et al. 2010; Welsh et al. 2012). Range‐testing is essential to optimize array configuration in order to achieve research objectives and accurately interpret animal detection results (Payne et al. 2010; Kessel et al. 2014b). However, range‐testing is sometimes under‐utilized by studies using passive acoustic technology and results rarely published when it is performed (Kessel et al. 2014b).

Managers at the Buck Island Reef National Monument (BIRNM), a federally protected marine area, have maintained a passive acoustic array since 2012 to better understand the spatial ecology of several keystone species that utilize habitat within the park. However, no formal range‐testing had been previously conducted, and therefore, variation in detection probability between receivers in different habitats remains unknown. Our objectives were to (1) estimate the probability of detection as a function of distance between the receiver and the tag throughout the current acoustic array at BIRNM and (2) determine how habitat, transmitter depth, wind speed, time of day, and sea surface temperature affect detection probability. We predicted that receivers in areas with less complex physical structure in deeper waters would have increased probability of detection and greater effective detection ranges.

## Methods

### Study site

**Figure 1:**
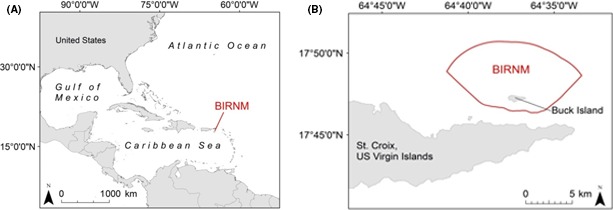
Location of Buck Island Reef National Monument (BIRNM). (A). Map showing the location of BIRNM within the Caribbean region. (B). The 73.4 km^2^ BIRNM with the boundary outlined in red.

**Figure 2:**
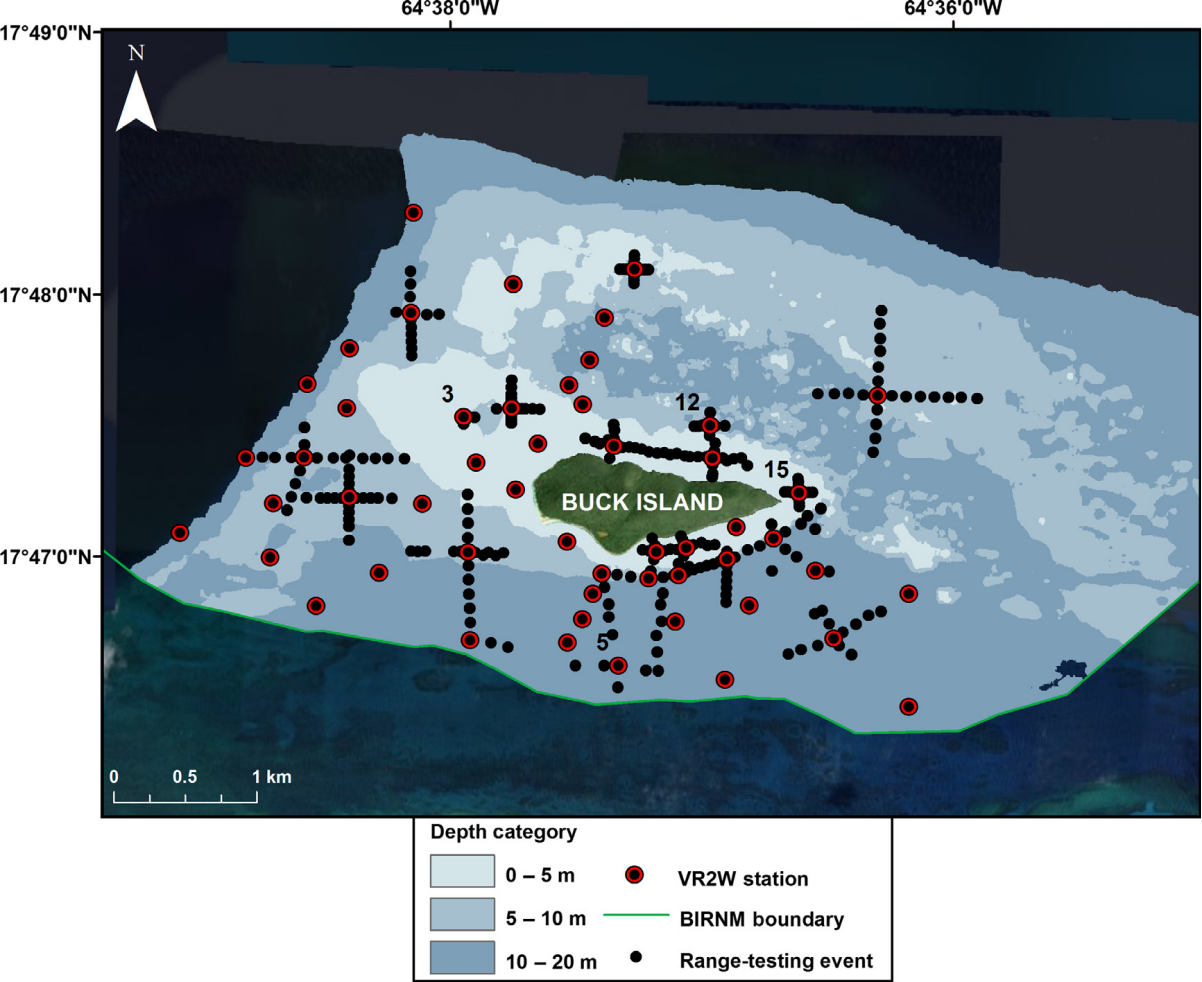
Bathymetry of the Buck Island Reef National Monument (BIRNM) Array. Binned bathymetry of array surrounding Buck Island. VR2W locations with numbers indicate receivers where preliminary 48‐h testing occurred.

**Figure 3:**
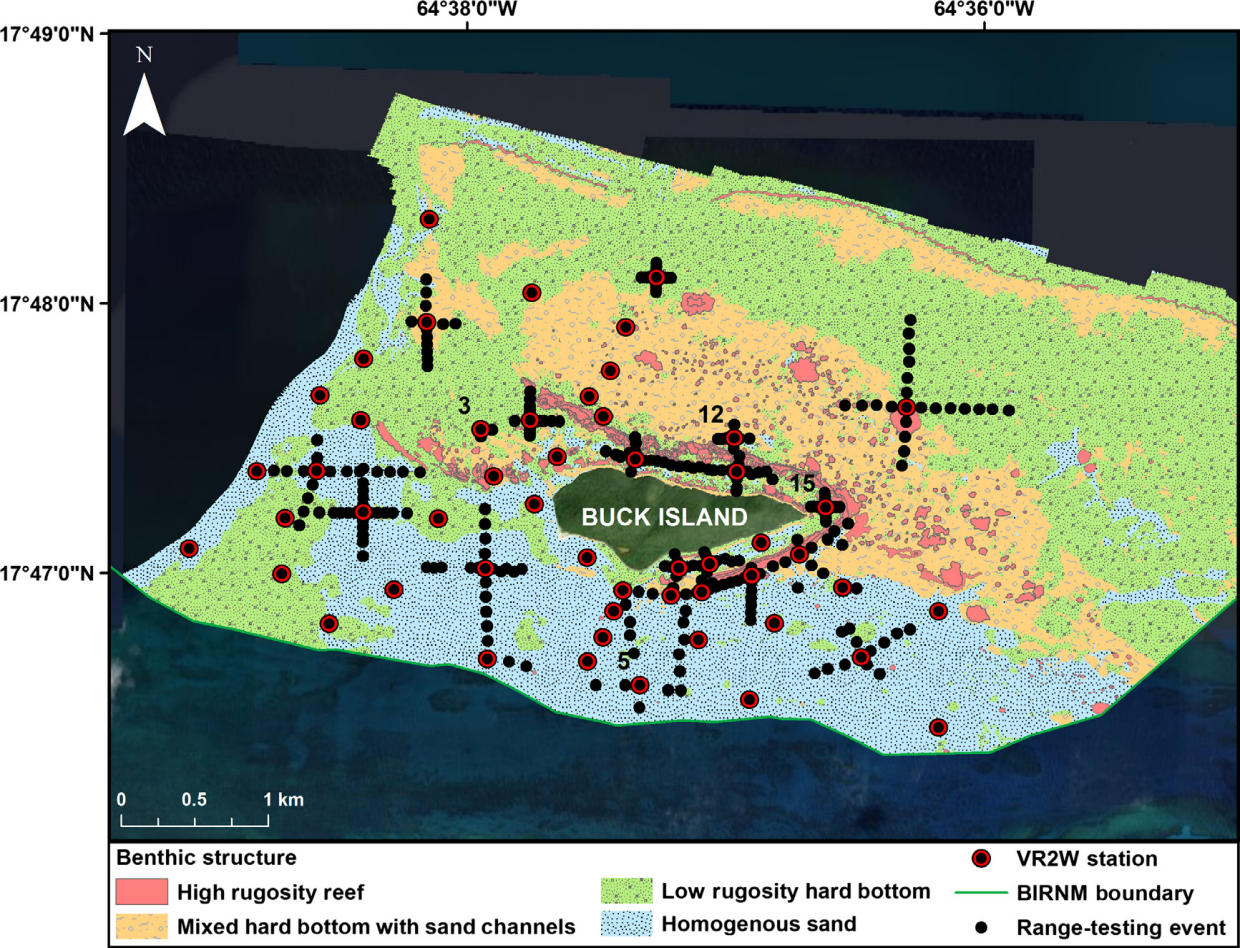
Benthic structure of Buck Island Reef National Monument (BIRNM) array. The location of VR2W receivers comprising the BIRNM (BIRNM) array dispersed throughout the various benthic structures along with the points where range‐testing events occurred. Benthic structure categories were binned according to similarity in rugosity.

Located 2.4 km northeast of the island of St. Croix, U.S. Virgin Islands, BIRNM encompasses 73.4 km^2^ of almost entirely submerged lands in addition to the 0.7 km^2^ uninhabited Buck Island (Hart et al. 2013; Fig. 1A and B). An emergent reef bank surrounds Buck Island creating a shallow 50‐150 m wide lagoon starting on the southern side and continuing counterclockwise to the northwest corner where it ends and becomes a series of isolated patch reefs. South/southwest of Buck Island, the reef bank slopes steeply to 12–15 m depths of mostly homogenous sand/sea grass beds sparsely interspersed with low lying reef‐rubble patches. Roughly 1 km north of Buck Island lies a submerged reef bank called the Buck Island Bar that runs east–west along the length of the coastal shelf (Bythell et al. 1993). In between Buck Island and the Buck Island Bar are densely clustered remnant stands of dead elkhorn coral (*Acropora palmata*) that rise to the surface from a depth of 9–15 m called “haystacks” (Mayor et al. 2006). West of the island, shallow sea grass beds (2–9 m) interspersed with aggregate reef patches gradually drop off into >15 m depths of fairly homogenous hard bottom substrate. The receiver locations are centralized around Buck Island and dispersed within the various habitats. There are, however, fewer receiver stations north of Buck Island due to the difficulty of navigating by boat within that area and the predicted limited effective detection range of receivers given the high density of complex coral structure. Habitat and depth at each receiver station are shown in Figures 2 and 3.

### Field methods

Range‐testing was conducted using four precision fixed delay transmitters (three Vemco V16‐4L and one V13‐1L, Bedford, Nova Scotia, Canada). One of the V16‐4Ls and the V13‐1L had 20 sec fixed delay intervals, whereas the two additional V16‐4L tags had 10 sec fixed delay intervals not including 3.5 sec to transmit the entire eight ping acoustic sequence (Matthew Holland, Vemco Customer Support, pers. comm.). Power output for the V13‐1L tag was 147 dB and for the V16‐4L tags was 152 dB. To preliminarily assess temporal differences in detection, we deployed the two 20‐sec delay interval tags near four receivers in distinct areas of the array. Tag start times were staggered 7‐sec apart to reduce the likelihood of signal collision and deployed for 48‐h periods at each of the four receivers. Tags were placed 25 and 40 m from receiver #12, 50 and 70 m from receiver #15, 50 and 75 m from receiver #03, and 100 and 150 m from receiver #05.

We conducted range‐testing in the fall of 2013 from September 6 to September 10 and from October 28 to November 14 and in the spring of 2014 from February 25 to March 5 and from April 28 to May 2. We focused on the first 19 receivers deployed as they were evenly distributed across the habitat classes covered by the array (Figs. 2, 3). Similar to the methods outlined by Sakabe and Lyle (2010) and Maljković and Côté (2011), we deployed range‐testing tags at predetermined distance intervals in each of the four cardinal directions from a receiver station which we termed an “event” (Sakabe and Lyle 2010; Maljković and Côté 2011; Figs. 2, 3). Range‐testing tags were left in place a minimum of one hour at each location. One hour was predicted to be the minimum amount of time allowable to accurately estimate detection efficiency at each location given the time constraints of the study (Matthew Holland, Vemco Customer Support, pers. comm.). In general, we spaced range‐testing events 100 m apart in relatively deep areas (>10 m) and 50 m in shallow habitats. We rarely deployed tags within ~500 m of each other in order to reduce the likelihood of signal collision (Matthew Holland, Vemco Customer Support, pers. comm.). However, in the interest of time, we occasionally performed two range‐testing events on the same receiver simultaneously by staggering the start times of the 20‐sec delay interval tags. The 10‐sec delay tags were never used for simultaneous range‐testing of a receiver.

**Figure 4:**
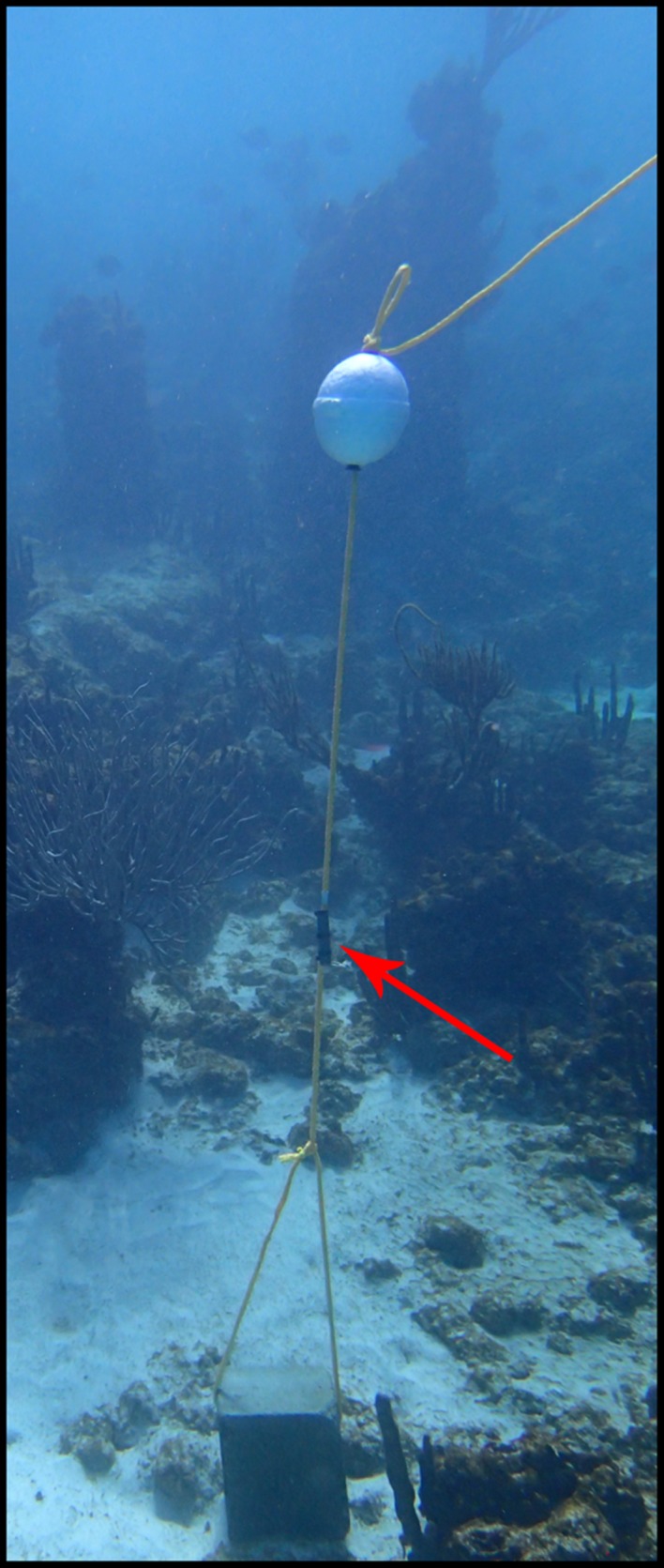
Range‐testing Tackle. Tackle used to deploy range‐testing transmitters at predetermined locations. Red arrow highlights the location of the range‐testing tag.

At the beginning of each event, a range‐testing tag was secured to a ~10 m polypropylene line 1 m above a cinder block anchor (Fig. 4). We secured each range‐testing tag to the line with the transmitting end pointed toward the surface of the water using zip ties. A 16.5‐cm Styrofoam float was secured 3 m above the transmitter to maintain vertical orientation, and a small six‐inch bright yellow surface float was attached to the end of the polypropylene line to aid in transmitter retrieval. At each preselected site, a free diver secured the tag and anchoring gear to avoid damaging live corals and ensure the tag was properly oriented. We recorded the start of a range‐testing event as the time the free diver was back on the boat and the end as the time when we arrived at the surface buoy to retrieve the tag. All clocks used to record time were synchronized to the local Atlantic Standard Time zone. National Park Service resource management staff performed downloads and maintenance of all receivers within the array.

### Data analysis

To analyze the preliminary 48‐h deployments, we separated tag deployment periods into 2‐min intervals and recorded detection (if ≥1 detection was recorded during a 2‐min interval; coded 1) or nondetection (if no detection occurred; coded 0). We then aggregated the number of successful detections by each hour of the day. Two‐minute time intervals were used to account for any variability in range‐testing tag transmission rate as well as mimic the typical delay intervals of tags attached to species of interest. We performed Fisher's exact test to identify significant differences between night and day in the number of successfully recorded detections at each of the four receivers.

To analyze the one‐hour range‐testing events, we uniquely labeled each event with a chronological identifier and the receiver station where the detection occurred. This was carried out because a single event could have been recorded by multiple receivers. If a range‐testing event was not recorded on any receiver, we used the nearest receiver to the event location. Similar to the preliminary analysis, we created 2‐min time intervals for each range‐testing event and recorded detection/nondetection within each interval.

We categorized 5 × 5 m habitat and depth cells in ArcGIS 10.0 (ArcGIS 10.0; ESRI 2010, Redlands, CA) using maps created by the National Oceanic and Atmospheric Administration (NOAA) based on Light Detection and Ranging (LiDAR), aerial orthophotos, and acoustic imagery (ESRI 2010; Costa et al. 2012). We used the dominant benthic structure field to create four habitat categories: homogenous sand, mixed hard bottom with sand channels, high rugosity reef, and low rugosity hard bottom. Categorizations were based on density of hard bottom structure and rugosity (surface complexity of physical structure). We binned bathymetry data into three depth classes: 0–5, 5–10, and >10 m. Using the Euclidean Distance tool in ArcGIS 10.0, we calculated the distance to the nearest receiver for each drop point with a 500‐m threshold (ESRI 2010). The Euclidean Allocation tool was also used to create a raster layer assigning each cell to the nearest receiver. We converted all raster files to ASCII text files and loaded them in RStudio to extract the values for each range‐testing event (R Development Core Team 2014). We averaged wind and water temperature data logged on NOAA station CHSV3 located in Christiansted Harbor ~5.5 km southwest of BIRNM for the duration of each drop interval. Detections were grouped into four time categories over a diel period: morning 06:00:00–10:59:59, afternoon 11:00:00–15:59:59, evening 16:00:00–19:59:59, and night 20:00:00–05:59:59. Finally, we tested for association among the independent variables using Pearson's chi‐square test, Cramer's V, variance influence factor, Spearman's rank correlation, association plots, and box plots.

In order to estimate the probability of detection for receivers within the array, we used generalized linear mixed models (GLMM) with binomial distribution implemented in RStudio with R version 3.2.2 (R Core Team 2015) and the package lme4 (Venables and Dichmont 2004; Bolker et al. 2009; Douglas Bates et al. 2015). Our independent variables were habitat (for receiver and tag), tag depth, distance to receiver, average sea surface temperature, and time of day. We used a logit link function with a binomial family structure and a binary response variable (*i.e*., detection/nondetection). The random effect in our models was the receiver where detections were recorded. We scaled distance between the receiver and the tag by subtracting the mean of all the distances and dividing by the standard deviation to achieve model convergence. We built the full model first with all of our variables included additively and then created subsequent models with reduced complexity by removing covariates. Pseudo‐R^2^ values were calculated for each model using the techniques outlined by Nakagawa and Schielzeth (2013) and Johnson (2014) (Nakagawa and Schielzeth 2013; Johnson 2014). We ranked models using Akaike information criterion (AICc) and difference between model AICc (ΔAICc). We compared model fit using Akaike weights, evidence ratios, and pseudo‐*R* ^2^ values. Models with ΔAIC_c_ <10 were considered to have some level of empirical support (Burnham and Anderson 2002).

Using the coefficients from the best‐supported model, we predicted the probability of detection based on stacked raster values of each cell for the BIRNM array map. For the nonrasterized covariates, we used the average wind speed and sea surface temperature for the entire data set and the mode of the time of day variable. Finally, we predicted probability of detection over distance given a receiver station's habitat type.

## Results

We performed 323 hour long range‐testing events excluding the preliminary 48‐h deployments. A total of 119,146 individual raw detections were recorded collectively on 33 different receivers throughout the array. There were 16,942 two‐minute time intervals coded as a “1” for detection for the one‐hour range‐testing events and 6,698 for the preliminary 48‐h range‐testing. Receiver detection ranges were somewhat limited across all habitat and depth classes, but also varied widely. Using the best‐supported model and holding the other variables constant, the average predicted probability of detection for tags 100 m away from a receiver throughout the entire array was 58.2% (95% CI: 44.0–73.0%). At 200 m from a receiver, predicted detection probability decreased to 26.0% (95% CI: 11.4–39.3). Predicted detection probability dropped to 0.2% (95% CI: 0.03–0.3%) 500 m away from a receiver.

**Figure 5:**
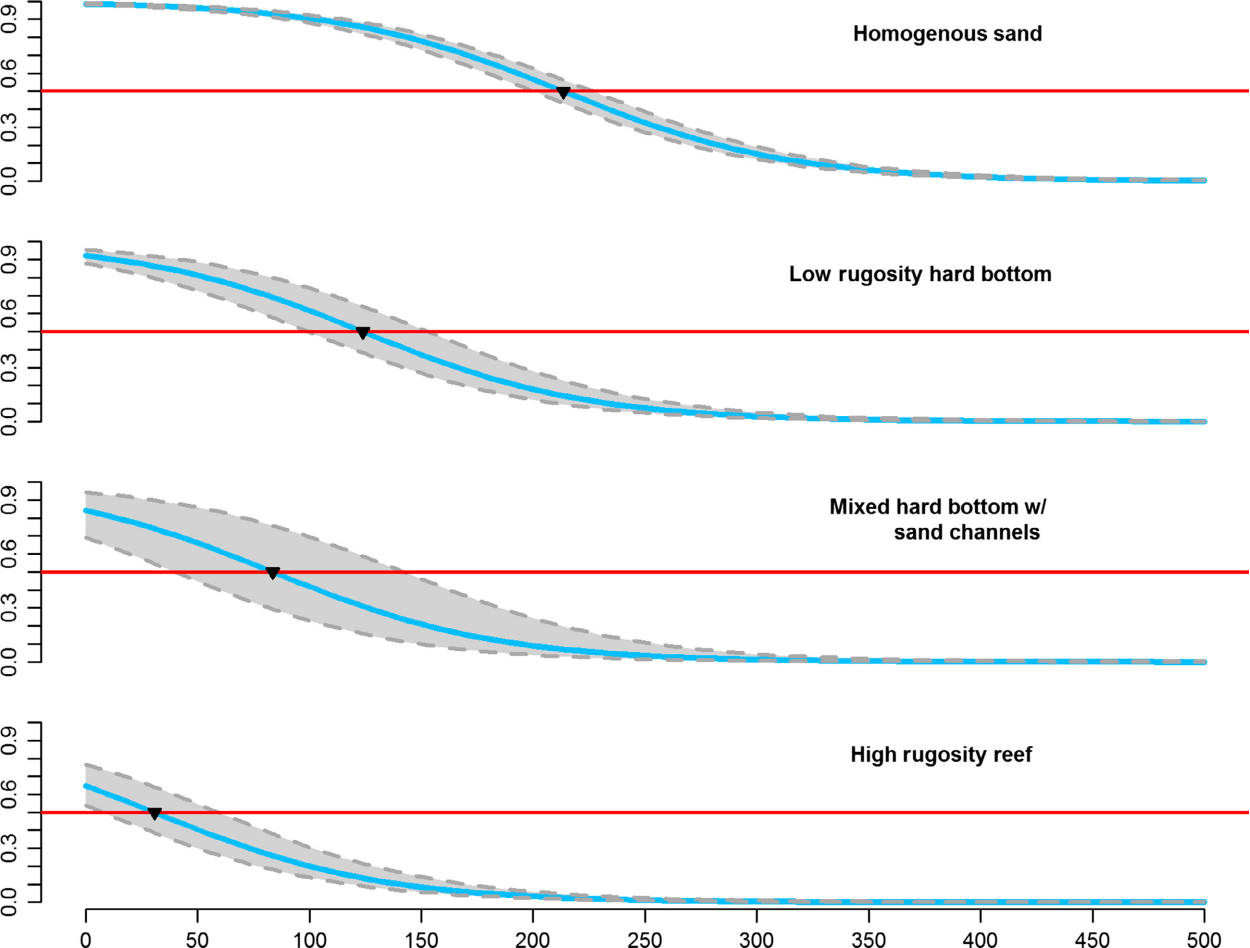
Predicted probability of detection over distance from receiver for each receiver habitat. Predicted probability of detection based on the full model for each benthic structure class (high rugosity reef, mixed hard bottom with sand channels, low rugosity hard bottom, and homogenous sand) using the average wind speed and sea surface temperature, and the 5‐10 m depth category during the afternoon time of day category. The red line denotes the 0.5 probability of detection for a transmitter (effective detection range). The black triangles reference the distance at which detection probability drops below 50%.

Model comparison indicated the full model best fit the data with the lowest AICc and 0.84 model weight (Table 1). Given that acoustic signal naturally attenuates over distance, it was expected that distance from receiver would be a highly important variable. The top 26 models (not all shown in Table 1) contained distance as a covariate, and the model containing only distance to receiver fit the data better than the model incorporating all other independent variables. The 5‐10 m depth range had the greatest negative coefficient estimate relative to the other depth categories. Effective detection range (distance at which ≥50% of detections are successfully recorded) in homogenous sand was 213.4 m (95% CI: 198.5–221.8 m), in low rugosity hard bottom, it was 123.9 m (95% CI: 95.9–152.3 m), in mixed hard bottom with sand channels, it was 83.7 m (95% CI: 36.5–142.7 m), and finally, it was 30.7 m (95% CI: 8.1–56.7 m) in high rugosity reef habitat. The predicted distance plots show the relationship between probability of detection and distance for each habitat class (Fig. 5).

**Table 1:** Model output and rankings

	Model	Parameters	AICc	Δ AICc	Weight	Marginal *R* ^2^	Conditional *R* ^2^
M.1	thab+rhab+dep+distsc+windsp+temp+tod (full)	16	14,143.92	0.00	0.84	0.58	0.86
M.2	thab+dep+distsc+windsp+temp+tod	13	14,147.17	3.25	0.16	0.53	0.88
M.3	thab+distsc+windsp+temp+tod	11	14,255.75	111.82	0.00	0.52	0.88
M.4	dep+distsc+windsp+temp+tod	10	15,022.75	878.83	0.00	0.54	0.86
M.5	rhab+distsc+windsp+temp+tod	11	15,189.14	1045.22	0.00	0.58	0.83
M.6	distsc	3	16,322.25	2178.33	0.00	0.49	0.81
M.7	thab+rhab+dep+windsp+temp+tod	15	22,788.55	8643.57	0.00	0.10	0.45
M.8	null	2	23,751.28	9607.36	0.00	0.00	0.40

A subset of generalized linear mixed models (GLMM) tested with the number of parameters in each model, Akaike information criterion (AICc), difference in model AICc (ΔAICc), the model weight, coefficient of determination for the fixed effect variables (marginal *R* ^2^), pseudocoefficient of determination for both fixed and random effects (conditional *R* ^2^); categorical variables include thab = transmitter habitat variable (low rugosity hard bottom, homogenous sand, high rugosity reef, and mixed hard bottom with sand channels), rhab = receiver habitat (low rugosity hard bottom, homogenous sand, high rugosity reef, and mixed hard bottom with sand channels) dep = depth class (0–5, 5–10, 10–15 m), time = time of day (06:00:00–10:59:59, 11:00:00–15:59:59, 16:00:00–19:59:59, and 20:00:00–05:59:59), continuous variables include dist = distance to receiver (m), windsp = wind speed (m/sec). Every model included receiver where detections occurred as an additive random effect.

**Figure 6:**
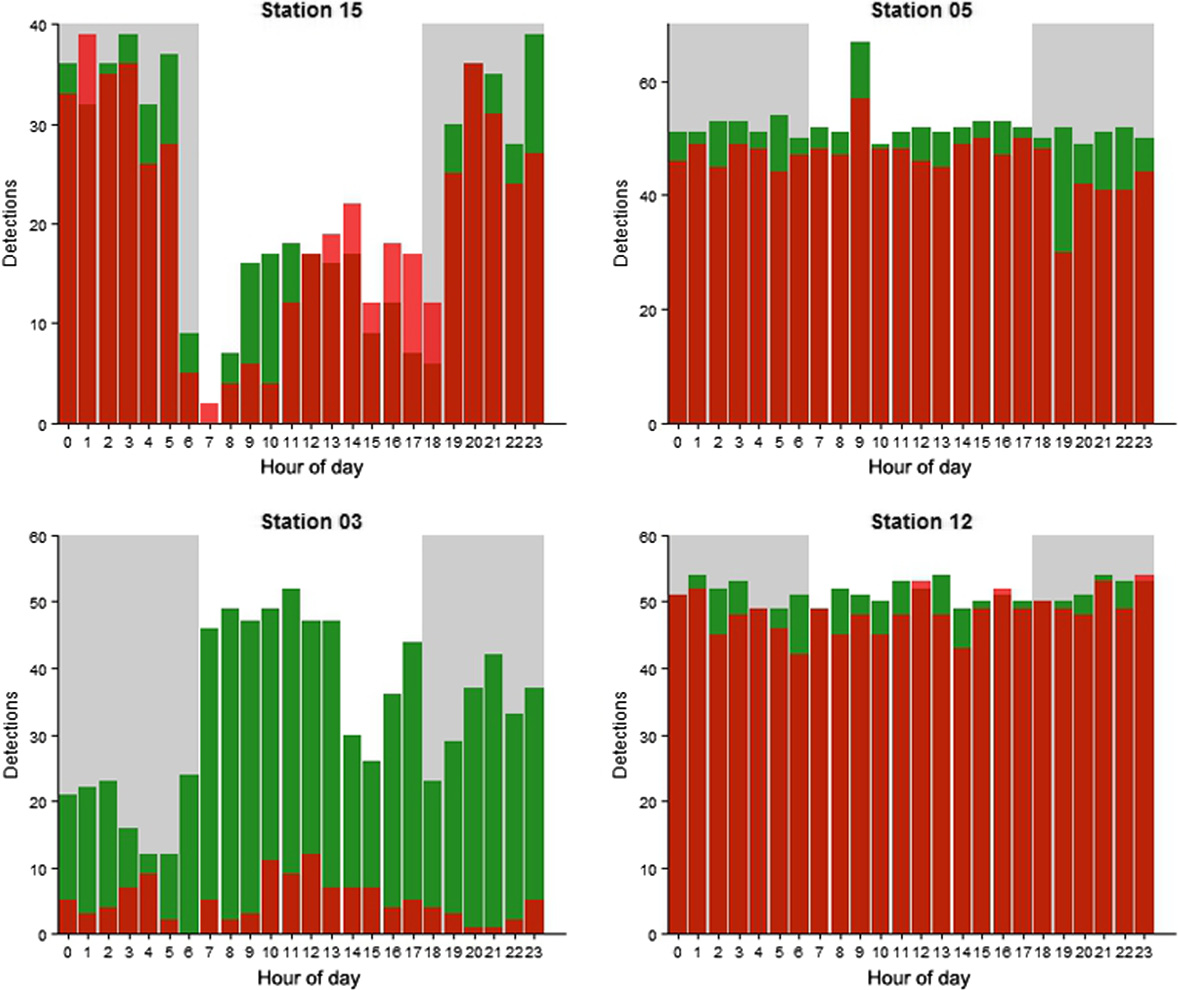
Preliminary 48‐h detection histories. Bar plots show the number of detections during each hour of the day for both transmitters deployed on each of the four receivers. Distance to receiver was staggered with the closer one denoted by green and the farther one red. Gray shading represents hours designated as night‐time.

Estimates for time of day coefficients indicate a greater probability of detection later into the evening and at night relative to events conducted in the morning (Table 2). The detection pattern at receiver #15 from the preliminary 48‐h deployed tags showed a similar trend with a significant decrease (*P* < 0.001) in the number of detections recorded during the day as opposed to the night based on Fisher's exact test. However, detection data from receiver #03 showed the opposite trend and receivers #05 and #12 had no significant difference in the number of detections between the night and day (Fig. 6).

**Table 2:** Model summary for fixed effect variables

Fixed effects	Estimate	SE	*z* value	Pr(>|*z*|)
Transmitter Benthic Structure				
Low rugosity hard bottom	1.71	0.06	27.96	<0.001
Mixed hard bottom w/sand channels	0.64	0.1	6.37	<0.001
High rugosity reef	1.05	0.08	13.63	<0.001
Receiver Benthic Structure				
Low rugosity hard bottom	−3.72	1.33	−2.79	0.005
Mixed hard bottom w/sand channels	−4.29	2.68	−1.6	0.109
High rugosity reef	−3.64	1.95	−1.87	0.062
Time of Day				
Afternoon	0.12	0.05	2.42	0.02
Evening	0.35	0.06	6.32	<0.001
Night	0.60	0.05	12.35	<0.001
Depth				
0–5 m	−0.54	0.1	−5.27	<0.001
5–10 m	−0.77	0.08	−9.82	<0.001

Table showing the estimate, standard error, *z*‐value, and *P*‐value for the categorical variables transmitter habitat, receiver habitat, time of day, and depth from the full model. Homogenous sand, morning, and 10–20 m were used as the reference categories for transmitter habitat, receiver habitat, time of day, and depth, respectively.

**Figure 7:**
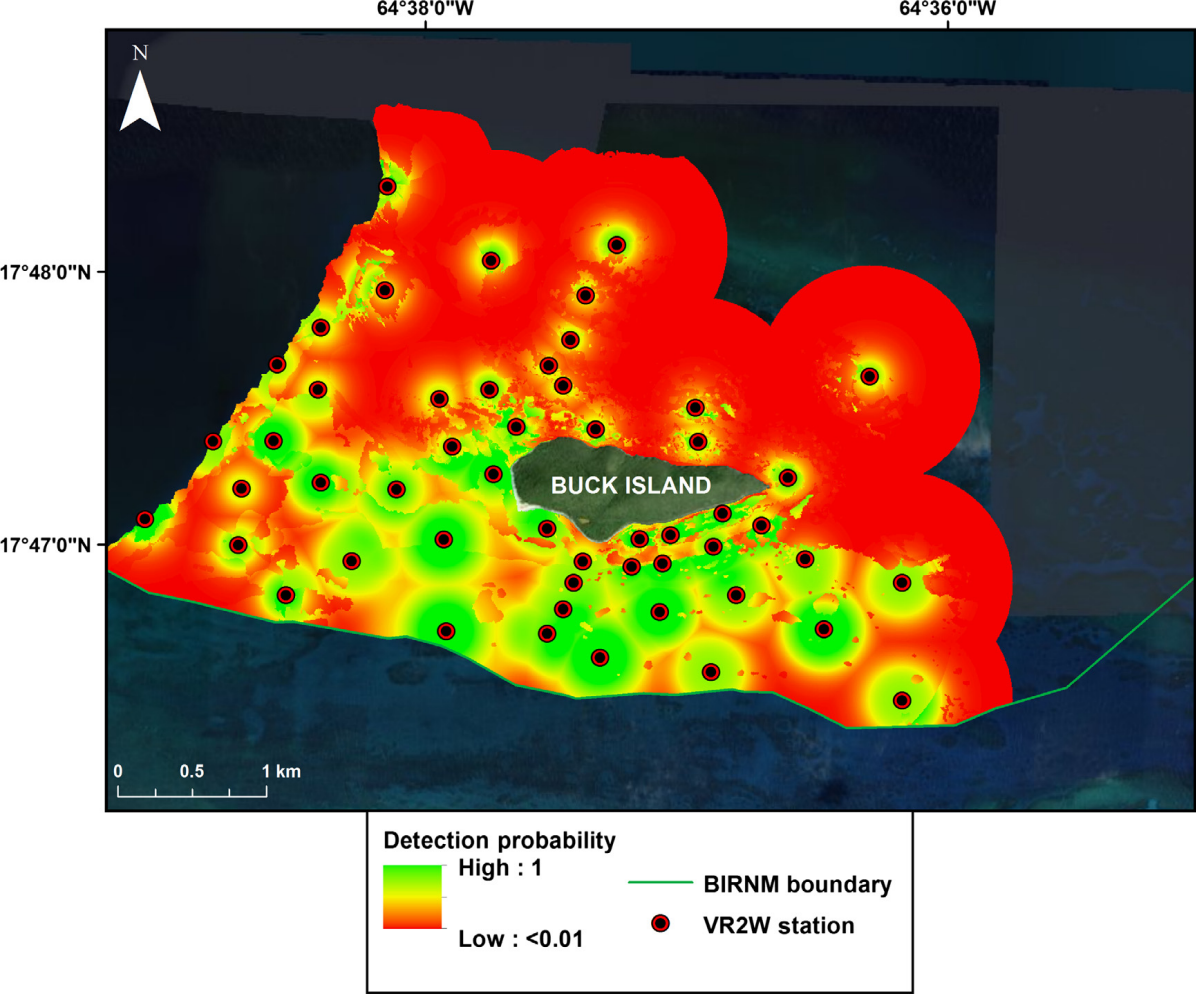
Predicted detection probability across the Buck Island Reef National Monument (BIRNM) array. Predicted probability of detecting an acoustic transmitter at BIRNM based on the full model.

**Figure 8:**
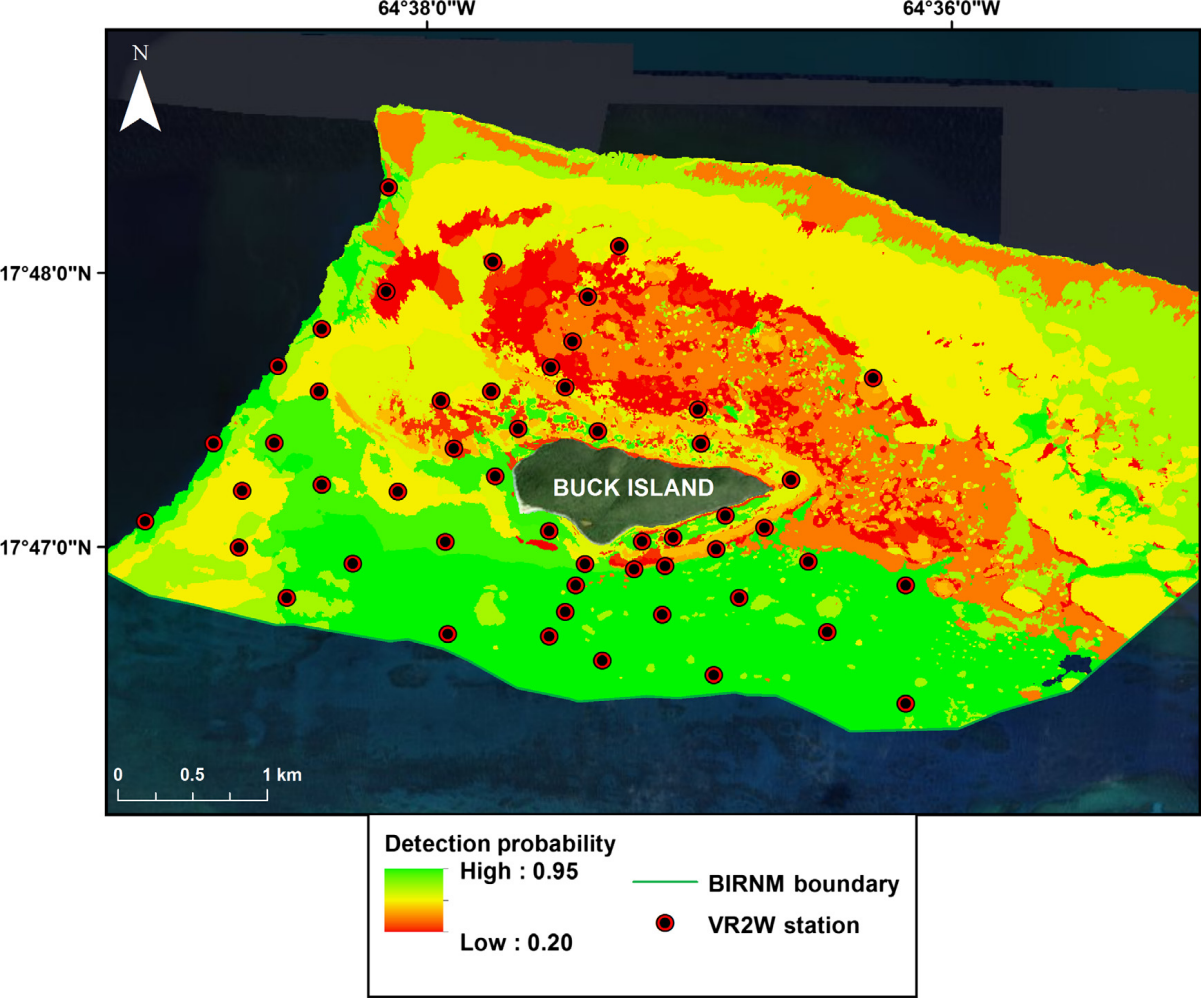
Predicted detection probability across the Buck Island shelf. Predicted probability of detecting an acoustic transmitter at Buck Island Reef National Monument (BIRNM) with distance from receiver held constant at 100 m. Map shows the relative suitability of a location for additional receivers given the habitat and depth of the area.

The predicted detection probability map for the array at BIRNM highlights the lack of coverage on the north/northeast side of Buck Island (where few receivers are located; Fig. 7). Overlap in receiver detection ranges appears unlikely in most areas throughout the array except for the line of four receivers that run perpendicular to Buck Island on the south side (Fig. 7). South of Buck Island has the largest area covered not only because of the greater number of receivers, but also their extended detection range. Holding distance to receiver constant and predicting across habitat and depth categories shows the highest probability of detection for receivers and tags located in the homogenous sand habitat in 10–20 m water depths south of Buck Island (Fig. 8). Detection probability appears limited north and northeast of Buck where the haystacks are most densely clustered.

## Discussion

Range‐testing results from studies in coastal coral reef habitat vary (Garla et al. 2006; Welsh et al. 2012; Cagua et al. 2013; Hazel et al. 2013; Garcia et al. 2014). Welsh et al. (2012) focused solely on range‐testing and found effective detection ranges of 90 m for Vemco V9‐1L tags (146 dB power output) along the base of a sloped coral reef habitat and 60 m at the reef crest in <2 m of water (Welsh et al. 2012). Hazel et al. (2013) reported effective detection ranges of 300 m along a relatively shallow reef flat for V16‐4L tags. Cagua et al. (2013) performed robust range‐testing at two sites on a platform reef in the Red Sea using fixed delay interval V13‐1x (153 dB) and V16P‐6H (160 dB) tags (Cagua et al. 2013). They deployed VR2W receivers at different distance intervals from a tag line for a period of 1–4 months and found an effective detection range of ~135 m (Cagua et al. 2013). The difference in methodology, equipment, and testing conditions is partially responsible for this variability, but study site habitat is likely a major factor as well (Cagua et al. 2013).

Our results indicate that the probability of detection is highly variable between receivers in different habitats. Predicted effective detection range was limited for receivers located in low rugosity hard bottom, mixed hard bottom with sand channels, and high rugosity reef. Relative to homogenous sand, these habitat types have a high density of physical structure that can impede and disrupt acoustic transmissions. Cagua, Berumen & Tyler found that even in reefs with low structural complexity, habitat features can highly influence detection probability (Cagua et al. 2013). Receivers south/southwest of Buck Island located primarily in homogenous sand habitat were predicted to have the largest effective detection ranges. Our results supplement Vemco's suggestion of placing receivers in areas where a clear line of site from the tag to receiver is easily achieved (Vemco 2015). Despite the overall constrained detection ranges for receiver stations north of Buck Island, areas between haystacks with primarily homogenous sand habitat surrounding them could provide additional coverage. Furthermore, receivers tethered higher above the sea floor may increase line of sight as VR2Ws are omnidirectional, thus minimizing signal attenuation caused by physical structure (Mathies et al. 2014). Finally, the predicted detection probability based on habitat and depth indicates that additional receivers north of the haystacks could potentially have relatively large effective detection ranges.

One limitation of our range‐testing method is that we used a 5 × 5 m raster to define benthic structure for the range‐testing event. Thus, receiver location alone does not fully capture the complexity of habitat types that may occur between a given receiver and transmitter. For instance, a receiver or transmitter located in homogenous sand habitat may be surrounded by high rugosity coral reef habitat that disrupts the acoustic signal. We used receiver habitat for our predictions because receiver location remains constant in our study, and therefore provides information about the probability of detecting tags on animals that may encounter several different habitat types within the detection range of a receiver. Also, we wanted to understand areas within BIRNM where detection probability of receivers would be maximized. A raster layer of rugosity could provide a more accurate metric of potential obstruction of acoustic signal in conjunction with an analysis technique that represents all the cells between the tag and receiver. However, more common LiDAR and other benthic mapping data layers can still provide researchers with the ability to preliminarily determine areas most likely to impede and reduce receiver detection range based on physical structure.

Depth can also be determined prior to deployment with the use of available bathymetry data. We predicted lower probability of detection for range‐tests performed in the shallowest depth category. Air bubbles near the surface caused by turbidity from wave action and rain increase acoustic signal attenuation (Gjelland and Hedger 2013). Our results show range‐testing events performed at 5–10 m depths had the greatest estimated negative coefficient suggesting decreased probability of detection for receivers within that depth range. However, this relationship was not specifically targeted in our study and the wide range of each depth category may have dampened the effect. Stratification within the water column due to currents or thermoclines can also cause variable detection ranges (Mathies et al. 2014). We therefore categorized depth because we did not assume a linear relationship beyond the interference high in the water column. Tides can also affect the probability of detection by altering the depth and orientation of a receiver and should be factored into range‐testing when relevant (Clements et al. 2005; Welsh et al. 2012; Mathies et al. 2014). Tidal and current fluctuations at BIRNM were not recorded during range‐testing. Historically, tidal amplitudes at BIRNM and around St. Croix have been small (<20 cm) with the largest fluctuations recorded being ~40 cm during spring tides. Using data from the nearby Christiansted harbor, we found the largest increase in mean sea level across all range‐testing was ~33 cm. Given that our shallowest receivers (<4 m) were anchored to the substrate by directly attaching them to the stem of sand screws, the effect of tidal fluctuations on receiver orientation was thought to be minimal. However, changes in currents throughout the water column often associated with tides have been shown to affect detection probability and should be considered especially if the array is expanded into deeper waters where receivers are commonly secured above the substrate on leashes with floats (Mathies et al. 2014). Transmitter depth can be unpredictable as well, depending on the vertical behavior of the species of interest and, despite the VR2W's omnidirectional coverage, may affect probability of detecting a transmitter. We tethered range‐testing transmitters 1 m off the seafloor to mimic observed juvenile marine turtle foraging behavior, the focus of a current U.S. Geological Survey study using the BIRNM array.

One complication of post‐deployment range‐testing, and most range‐testing in general, is correlation among independent variables. We used association plots, Pearson's chi‐square test, and Cramer's V statistic to assess correlation between tag and receiver habitat and depth. Chi‐square values indicate correlation between both habitat categories and depth. The strength of correlation indicated by Cramer's V statistic was greater for tag habitat (0.35) as opposed to receiver habitat variable (0.22). Association plots for both receiver and transmitter habitat showed range‐testing was performed less frequently in low rugosity hard bottom habitat at deeper depths, but more in the shallowest depth class. Habitat variables were also correlated with one another and had a Cramer's V of 0.38. However, the variance inflation factor was low enough (<3) to indicate that multicollinearity does not drastically influence our predictions. No significant correlation was found among the other independent variables.

Time of day, wind speed, and sea surface temperature variables were included to help control for multi‐season range‐testing. The methods used provide a snapshot of the spatial extent of a passive acoustic array, but it is critical to note they do not account for temporal variations. Areas where overlap of detection ranges between receivers occurs, such as the line of four perpendicular receivers south of Buck, may not provide the same coverage consistently throughout the year. Long‐term control tags are necessary for discerning behavioral shifts from temporal fluctuations in receiver detection range (Payne et al. 2010). The longer 48‐h intervals used in the preliminary analyses indicate possible temporal fluctuations in detection probability. Detection data from receiver #03 showed a significant decrease in the number of detections at night, a trend consistent with previous research and possibly explained by biological noise (Payne et al. 2010; Cagua et al. 2013). However, receiver #15 had the opposite trend and receivers #12 and #05 had no significant difference between the number of detections recorded during the night and day. Increased noise interference from concessioner boats motoring up to buoys in close proximity to receiver #15 twice a day for snorkeling tours could possibly contribute to this pattern. Receiver #15 is also one of the more shallow receiver locations (~3.3 m) and is located in area with little protection from wind and wave action. Despite little explanatory power, the preliminary 48‐h results suggest the need to consider habitat when deploying sentinel tags in a complex environment. Variability in temporal fluctuations may not be uniform across the array.

Animal behavior and tag delay interval (transmission rate) will ultimately affect the probability of detection. We used two‐minute time intervals to control for variability in transmission rate among range‐testing tags as well as provide coherent interpretation for tags deployed on marine turtles with 30‐90 sec transmission delay intervals. Less mobile species such as queen conch (*Lobatus gigas*) may have an increased probability of detection further away from a receiver due to a greater number of transmissions than a more mobile species that is in the same area for a limited amount of time. Conversely, constrained detection ranges may not be problematic for highly mobile species such as some species of fish and marine turtles that potentially cover a large area in a relatively short time span. Decreased detection ranges allow for a more accurate interpretation of location as well. However, species with limited movement patterns will have a decreased chance of detection if they primarily inhabit areas where coverage is sparse such as north/northeast of Buck Island. Finally, tag type should be considered when performing range‐testing. We used two of the larger high powered tags offered by Vemco, and due to time constraints, we deployed tags based on proximity of location from the previous range‐testing event. Our predicted effective detection ranges therefore may be constrained for higher powered tags such as V16s and larger than expected for tags with lower output as used by some researchers in the BIRNM array. However, How and de Lestang (2012) show that the relationship between power output and distance should not be assumed linear as it can be influenced by environmental variables (How and de Lestang 2012).

The maps showing predicted probability of detection throughout BIRNM highlight areas where additional receivers might offer the best coverage. However, areas where receiver detection range is constrained, in the haystacks for instance, should not be abandoned given the coarse categorization of habitats. Overall, receivers placed in the haystacks may have constrained effective detection ranges, but can still provide necessary coverage in that area by being placed in locations where line of sight to the receiver can be maximized. Besides informing additional receiver locations, our results can also help more accurately determine residency time for tagged individuals by weighting detections based on the receiver's effective detection range where they occurred.

## Conclusions

As passive acoustic technology continues to improve, the cost of existing equipment will likely decrease, making it more readily available to researchers and resource managers and enabling larger arrays. Large, long‐term arrays can provide important movement data and residency information on a number of different species, but it is important to understand how receivers function in their environment in order to meet or revise research objectives. The Caribbean coastal environment is dynamic with numerous variables that can potentially affect receiver detection range and should be taken into consideration when configuring an array.

Here, we range‐tested a relatively low‐cost passive acoustic array system, discerned factors influencing effective detection range, and developed a method for estimating array coverage at BIRNM. Our results indicate that deep habitat with minimally rugose substrate provides the greatest effective detection range and increased probability of detection. Our study revealed that array coverage north/northeast of Buck Island is poor at best; additional receiver stations in this part of BIRNM would be needed to provide increased coverage of that area. Furthermore, our study identifies variables affecting detection range that should be monitored throughout the course of the study with long‐term control tags. Understanding how each variable affects probability of detection or effective range of detection may not be necessary for every study design, but being aware of potential variations in detection range or lack of coverage is crucial for designing and implementing ecological studies based on an acoustic array.

Postdeployment range‐testing is obviously constrained by the existing receiver station locations. However, range‐testing should not be abandoned once receivers have already been deployed, especially when a study is long term. Our results not only provide important information for future placement of additional receivers at BIRNM, but also highlight some of the constraints in using passive acoustic technology in a Caribbean coastal environment.

## Data Accessibility

Data and R scripts will be held at 3321 College Ave. Davie, FL 33314 and will be uploaded to a USGS server if manuscript is accepted for publication.

## Conflict of Interest

None declared.
